# Quantitative diagnostic method to detect Gardnerella vaginalis by droplet digital PCR

**DOI:** 10.1016/j.plabm.2025.e00499

**Published:** 2025-08-22

**Authors:** Yong-Zhuo Zhou, Yun-Hu Zhao, Yan-Lan Chen, Wei-Zhen Fang, Bi-Si Liang, Xu-Guang Guo, Chao-Hui Duan, Hui-Ling Hu

**Affiliations:** a Department of Clinical Laboratory, Sun Yat-sen Memorial Hospital, Sun Yat-sen University, Guangzhou, 510120, China; b Institute of Gerontology, Guangzhou Geriatric Hospital, Guangzhou Medical University, Guangzhou, 51000, China; c State Key Laboratory of Respiratory Disease, Guangzhou Geriatric Hospital, Guangzhou Medical University, Guangzhou, 51000, China; d Collaborative Innovation Center for Civil Affairs of Guangzhou, Guangzhou, 51000, China; e Department of Clinical Laboratory Medicine, Guangzhou Geriatric Hospital, Guangzhou Medical University, Guangzhou, 51000, China; f Department of Laboratory Medicine, Guangdong Provincial People's Hospital (Guangdong Academy of Medical Sciences), Southern Medical University, Guangzhou, 510080, China; g Guangzhou Xiaoqi Biotechnology Co., LTD, Guangzhou, 510699, China; h Guangzhou Archean Gene S&T Co.,LTD, Guangzhou, 510000, China

**Keywords:** Droplet digital PCR, *Gardnerella vaginalis*, Q*uantitative* testing

## Abstract

**Background:**

Nucleic Acid Amplification Tests (NAAT) remain one of the most reliable methods for pathogen identification. Given the high false-negative rates associated with traditional staining and microscopic examination, the time-consuming nature and low sensitivity of bacterial culture methods, as well as the inability of conventional NAAT to achieve absolute quantification.

**Methods:**

To achieve rapid and quantitative detection of *Gardnerella vaginalis*, we selected the 23S rRNA gene as the target for identification and developed a droplet digital PCR detection method.

**Results:**

The entire detection process can be completed within 92 min, demonstrating high efficiency. The sensitivity reached 4.4 pg/μL, and no positive droplets were detected in experiments involving eight negative control pathogens, confirming high specificity. Additionally, the ddPCR assay for *Gardnerella vaginalis* exhibited excellent repeatability, with a calculated coefficient of variation of 1 %.

**Conclusion:**

The ddPCR detection technology demonstrates characteristics such as absolute quantification, high sensitivity, high specificity, and high reproducibility for *Gardnerella vaginalis*, showing promise as an excellent testing platform. This advancement could provide a more scientific basis for clinical diagnosis and treatment.

## 1 Introduction

*Gardnerella vaginalis*, a pathogenic bacterium isolated from vaginal secretions of female patients, serves as the primary causative agent of bacterial vaginosis [1]. Beyond bacterial vaginosis, *Gardnerella vaginalis* is implicated in multiple gynecological and urogenital disorders, including cervicitis, spontaneous abortion, postoperative infections, and urinary tract infections in females, as well as prostatitis in males [[2], [3], [4], [5]]. These clinical manifestations underscore its substantial impact on public health systems.

The current clinical diagnosis of bacterial vaginosis primarily relies on microscopic examination of vaginal secretion smears using gram staining, where the detection of clue cells serves as one of the diagnostic indicators. However, *Gardnerella vaginalis* exhibits Gram-variable staining characteristics, often appearing as pleomorphic bacilli with inconsistent Gram-positive/negative properties [6]. Consequently, factors such as sampling techniques, smear preparation quality, and staining conditions may compromise the detection rate of clue cells, potentially leading to false negatives. Traditional culture methods are time-consuming and exhibit low sensitivity, particularly in patients with genitourinary infections where empirical antibiotic use might suppress pathogen growth, further increasing the risk of missed diagnoses [7]. Although conventional nucleic acid amplification tests have been explored for *Gardnerella vaginalis* detection, their clinical utility is limited by insufficient sensitivity and inability to provide quantitative pathogen analysis. These limitations underscore the necessity to establish a more reliable detection method for *Gardnerella vaginalis* with enhanced accuracy and quantitative capabilities.

Digital PCR, as a refinement of qPCR technology, enables more precise nucleic acid detection with simplified and intuitive data analysis. In recent years, multiple studies have demonstrated the significant potential of droplet digital PCR (ddPCR) in clinical diagnosis and therapeutic monitoring [8,9]. Given the technical advantages of ddPCR and the absence of reported applications for *Gardnerella vaginalis* detection, this study aims to establish a ddPCR method targeting *Gardnerella vaginalis*. We systematically evaluated its specificity, repeatability, and sensitivity to assess whether ddPCR could serve as a novel diagnostic platform for this pathogen.

## 2 Materials and methods

### 2.1 Standard bacterial strains and other bacterial strains

The standard strain of *Gardnerella vaginalis* (ATCC 14018) was sourced from the Department of Laboratory Medicine, Sun Yat-sen Memorial Hospital. Eight species of negative control bacteria were collected from Sun Yat-sen Memorial Hospital, Sun Yat-sen University, with *Mycoplasma genitalium* and *Chlamvdia trachomatis* procured from positive samples in the PCR Laboratory, while the remaining strains were isolated from clinical specimens in the Microbiology Department. All bacterial strains used in this study are listed in Table 1.

**Table 1:** Bacterial strains used in this study.

*Gardnerella vaginalis ATCC*14018
*Lactobacillus*
*Enterococcus faecalis*
*Enterococcus faecium*
*Pseudomonas aeruginosa*
*Proteus mirabilis*
*Staphylococcus aureus*
*Mycoplasma genitalium*
*Chlamvdia trachomatis*

### 2.2 Nucleic acid extraction

We performed nucleic acid extraction using the pre-packaged nucleic acid extraction kit (Da'an Gene Co., Ltd., Guangzhou) on the Smart32 system. The procedure strictly followed the manufacturer's protocol, with detailed steps as follows:

(1) Inoculated preserved strains of *Lactobacillus*, *Enterococcus faecalis*, *Enterococcus faecium*, *Pseudomonas aeruginosa*, *Staphylococcus aureus*, and *Proteus mirabilis* onto blood agar plates for 18–24h incubation at 37 °C;
(2) Suspended bacterial colonies in normal saline to achieve 3–4 McFarland standard suspensions;
(3) Processed *Chlamydia trachomatis* and *Mycoplasma genitalium* directly from bacterial preservation medium using identical extraction methodology;
(4) Added 20 μL Proteinase K solution to each well in Row 1 of the pre-packaged extraction plate;
(5) Transferred 200 μL bacterial suspensions to corresponding Row 1 wells with appropriate strain labeling;
(6) Executed extraction protocol: Bead mixing (1min) → Bead binding at 70 °C (15min) → First wash (1 min) → Second wash (1 min) → Third wash (1min) → First elution (2 min) → Second elution at 70 °C (2 min) → Magnetic bead discard (1 min);
(7) Final nucleic acid extracts about 50 μL in Row 6 wells were preserved for subsequent use.

### 2.3 Primer design and synthesis

In this study, the 23S rRNA gene of *Gardnerella vaginalis* (serial number LC774556.1)was selected as the target gene [10]. The base sequences were searched in NCBI (https://www.ncbi.nlm.nih.gov/) and six sets of primers were designed separately. The primers were synthesized by Shanghai Biological Engineering Co., Ltd. The sequences of the 23S rRNA gene and the primer sequences used in study are shown in Table 2, Table 3.

**Table 2:** 23S rRNA gene sequence of *Gardnerella vaginalis*.

GGTAGACAGGACCGATGAAGGACGTGACGGGCTGCGATATGCCTCGGGGAGCTGCCGAGTGGGCTTTGATCCGAGGATTTCCGAATGGGGAGACCCGGCCACTGTTATGGGTGGTCACCACAGTTTTGGGGGGGGGTCCCCAGGAAATGGAACCTCCCCATTCCCGGCGGGAAAGGATCTCCCGGGATTATGGCCAACCAAAACGGAATCAGGTTAACCCAATACCTGGGGATACCCGCCGGGGGTGGCTATTTCGGGGCCGGGGAATTGATGGTC

**Table 3:** Primers for the *Gardnerella vaginalis* 23S rRNA gene.

Primers	Sequence(5’ →3′)	Length (bp)
23S rRNA-1 upstream	GGACCGATGAAGGACGTGAC	20
23S rRNA-1 downstream	ATGTTTCAGTTCCCCTGCGT	20
23S rRNA-2 upstream	TCCGAGGATTTCCGAATGGG	20
23S rRNA-2 downstream	TGTTTCAGTTCCCCTGCGT	19
23S rRNA-3 upstream	CAGGACCGATGAAGGACGTG	20
23S rRNA-3 downstream	ATCCTCGGATCAAAGCCCAC	20
23S rRNA-4 upstream	GAGTGGGCTTTGATCCGAGG	20
23S rRNA-4 downstream	TGTTTCAGTTCCCCTGCGTA	20
23S rRNA-5 upstream	ATTTCCGAATGGGGAGACCC	20
23S rRNA-5 downstream	GATGTTTCAGTTCCCCTGCG	20
23S rRNA-6 upstream	GAGACCCGGCCACTGTTATG	20
23S rRNA-6 downstream	AGATGTTTCAGTTCCCCTGCG	21

### 2.4 Primer screening analysis and droplet digital PCR

Real-time fluorescence quantitative PCR was performed by using *Gardnerella vaginalis* 23S rRNA gene and corresponding six sets of primers. The reaction system and amplification procedure are shown in Table 4, Table 5. The reaction procedure is the same as the ddPCR amplification conditions except for the melting curve part. Compare the amplification results of 6 sets of primers, and select the optimal primers for ddPCR experiments based on the amplification efficiency and the melting curve. In this study, droplets were initially generated using the DG32 Droplet Generator (Pilot Gene Technologies Co.,Ltd.). Subsequently, chip-based digital PCR was performed on the TC1 Digital PCR System for amplification. The reaction droplets were then scanned with the CS7 Biochip Reader to quantify amplified positive droplets [11,12].

**Table 4:** Composition of the reaction mix.

	Components	Volume
Reaction mix	Tris–HCl(20 mM PH8.6)	12.5 μl
	KCl(8 mM)	
	MgCl_2_(6.0 mM)	
	Tween-20(0.1 %)	
	Betaine(1 mM)	
	1.6 mM dNTP	
DNA template		6 μl
Primer mix	upstream(0.2 μM)	1 μl
	downstream(0.2 μM)	
Abstract Taq DNA Polymerase		1 μl
ddwater		4.5 μl
Total volume		25 μl

**Table 5:** Reaction procedure for qPCR.

Step	Temperature	Time	cycles
Initial Denaturation	50 °C	120s	–
	95 °C	150s	–
Denaturation	94 °C	35s	40
Annealing&Extension	60 °C	35s	40
Melting Curve			
- Ramp-up	95 °C	15s	–
- Ramp-down	60 °C	60s	–
- Final Denaturation	95 °C	15s	–
- Cooling	60 °C	15s	–

### 2.5 Specificity of ddPCR for *Gardnerella vaginalis*

The genomic DNA of standard strains of *Gardnerella vaginalis* and genomic DNA of 8 negative control strains were amplified simultaneously by ddPCR reaction program, and the detection signals of droplets were analyzed to evaluate the specificity of primers for *Gardnerella vaginalis*.

### 2.6 Sensitivity of ddPCR for *Gardnerella vaginalis*

The concentration of nucleic acid was detected by the Thermo Scientific Nanodrop 2000 microspectrophotometer. The extracted nucleic acid stock solution was diluted by gradient with ultrapure water for 4 times to obtain 5 nucleic acid concentrations, and ultrapure water was used as negative control. Then, the ddPCR amplification reaction was performed, and the positive droplets were observed to evaluate the sensitivity of ddPCR to *Gardnerella vaginalis*.

### 2.7 Repeatability of ddPCR for *Gardnerella vaginalis*

The genomic DNA of *Gardnerella vaginalis* standard strain was amplified for three times, the number of positive droplets was compared, and the coefficient of variation was calculated to evaluate the reliability of primers for droplet digital PCR.

## 3 Result

### 3.1 Primer screening test and melting curve analysis

**Fig. 1:**
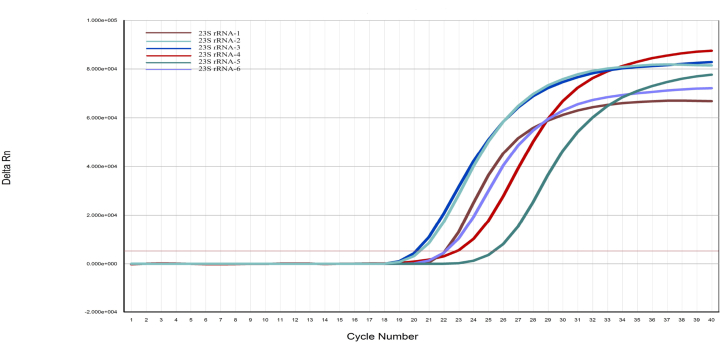
The primer of the qPCR screening experiment.

**Fig. 2:**
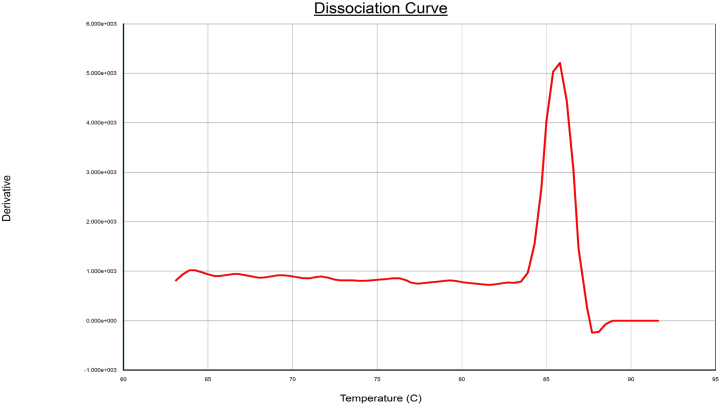
The melting curve of primer 23S rRNA-3.

In this study, primer screening tests were performed by qPCR. The optimal set of primers is screened according to the amplification efficiency and the melting curve. The results showed that primer 23SrRNA-3 had the highest amplification efficiency, with an amplification reaction inflection point appearing in the 20th cycle and reaching the peak amplification reaction platform in the 32nd cycle(Fig. 1). The qPCR melting curves of all 6 sets of primers showed a single peak, indicating that the reaction did not involve non-specific amplification or primer dimers. Only the melting curve of primer 23SrRNA-3 is shown here (Fig. 2). But the amplification efficiency was all lower than that of 23S rRNA-3, 23S rRNA-3 was selected for subsequent experiments.

### 3.2 Specificity of ddPCR for *Gardnerella vaginalis*

**Fig. 3:**
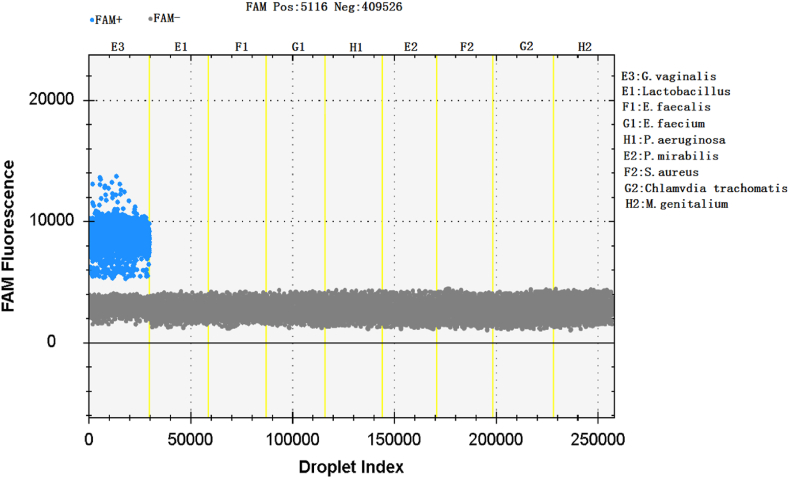
Specificity of ddPCR for the amplification of *Gardnerella vaginalis*.

The genomic DNA of *Gardnerella vaginalis* and 8 negative control bacteria were simultaneously amplified by ddPCR, and the results showed that only *Gardnerella vaginalis* was detected as positive droplets, while none of the negative control bacteria were detected as positive droplets. It shows that ddPCR detection of *Gardnerella vaginalis* 23SrRNA-3 gene has good specificity and did not display cross-reactivity with non-target genes (Fig. 3). The horizontal axis in the image represents the droplet index, and the vertical axis represents the fluorescence intensity detected by the FAM channel. Negative and positive droplets are represented in gray and blue, respectively.

### 3.3 Sensitivity of ddPCR for *Gardnerella vaginalis*

**Fig. 4:**
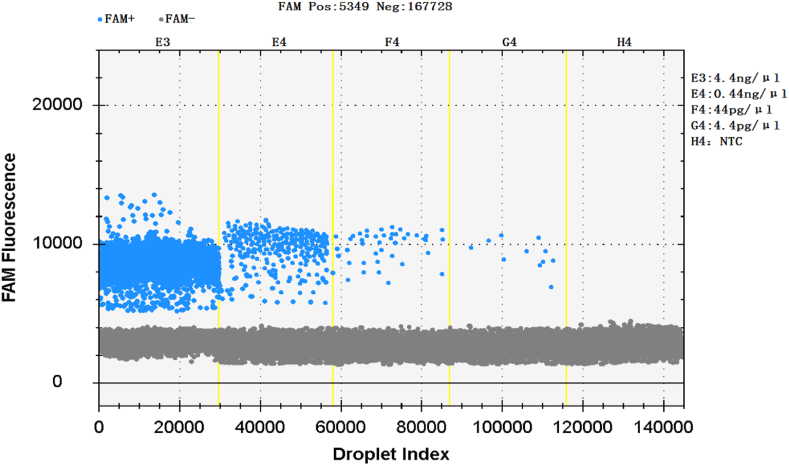
Sensitivity of ddPCR for the amplification of *Gardnerella vaginalis*.

The concentration of nucleic acid of *Gardnerella vaginalis* was measured to be 4.4ng/μ L by using microspectrophotometer, and0.44 ng/μL, 44 pg/μL, 4.4 pg/μL and 0.44 pg/μL were obtained after 4 times of dilution. The experimental results show that the sensitivity can be as high as 4.4 pg/μL, and the corresponding biochip reader CS7 reading is 0.35copies/μL, indicating good sensitivity (Fig. 4). Ten repeatability tests were performed for each of the two nucleic acid concentrations of 4.4 pg/μL and 0.44pg/μ. The results showed that all ten repeatability tests of 4.4 pg/μL were positive, and nine of the ten repeatability tests of 0.4 pg/μL were negative and one was positive.

### 3.4 Repeatability of ddPCR for *Gardnerella vaginalis*

**Fig. 5:**
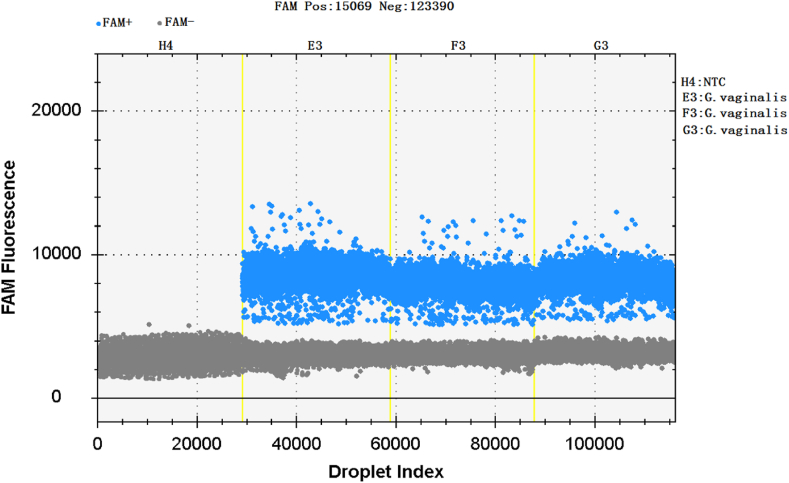
Repeatability of ddPCR for the amplification of *Gardnerella vaginalis*.

Three repeated experiments for the standard strain of *Gardnerella vaginalis* were performed by ddPCR. The results showed that the number of positive droplets were 575, 580, and 570 copies/μL, respectively. The coefficient of variation(CV) was calculated to be 1 %. The detection were stable, indicating good reproducibility (Fig. 5).

## 4 Discussion

*Gardnerella vaginalis* infection presents as vaginitis with non-specific symptoms similar to those of other pathogenic bacteria infections. Failure to treat in time after infection or incomplete treatment can lead to serious consequences, especially in pregnant women, which can lead to miscarriage or postpartum endometritis. In severe cases, it can also lead to sepsis, perirenal abscess, and cystitis [[13], [14], [15], [16], [17]]. The ddPCR method established in this study achieved absolute quantitative detection of *Gardnerella vaginalis* without relying on standard curves. This made it possible to correlate *Gardnerella vaginalis* with the severity of bacterial vaginosis and the efficacy analysis. Compared with the traditional culture method, ddPCR can shorten the detection time significantly, and the sensitivity is significantly better than qPCR (usually 1 copy/μL). This advantage made it suitable for screening of low-load infections or asymptomatic carriers.

Our experimental data shows that ddPCR can stably detect low concentrations of *Gardnerella vaginalis* in simulated clinical samples, and has no cross reactivity with common vaginal commensals and pathogenic bacteria. This high specificity is attributed to primers designed for the conserved gene 23S rRNA targeting *Gardnerella vaginalis*. DdPCR detection of *Gardnerella vaginalis* has good reproducibility with a CV of 1 %. This experiment is relatively fast in detection, with an amplification process that can be completed in 40 min. The entire detection process takes 92 min, which can be slightly shortened compared to ordinary PCR detection time, which is beneficial for rapid clinical diagnosis. Given the above advantages, ddPCR can be used to determine the expression and copy number variation analysis of target genes. However, limitations are also inevitable. Although ddPCR has high sensitivity, its detection cost is still higher than conventional qPCR, and its throughput is lower. In addition, the preprocessing steps of vaginal samples may affect quantitative accuracy, such as DNA extraction efficiency. Subsequent research can optimize the extraction scheme or introduce internal reference gene for correction.

Our study shows that ddPCR detection of *Gardnerella vaginalis* is superior to traditional methods, with the characteristics of high specificity, high sensitivity, high reproducibility, and absolute quantification. In the future, a large number of samples and different types of samples can be collected for further validation experiments to support the detection capability of this method, which may become one of the clinical diagnostic tools.

## CRediT authorship contribution statement

**Yong-Zhuo Zhou:** Conceptualization, Data curation, Formal analysis, Resources, Writing – original draft, Writing – review & editing, Investigation, Project administration. **Yun-Hu Zhao:** Validation, Resources, Methodology, Formal analysis. **Yan-Lan Chen:** Validation, Resources, Investigation. **Wei-Zhen Fang:** Visualization, Validation, Supervision, Formal analysis. **Bi-Si Liang:** Validation, Methodology. **Xu-Guang Guo:** Visualization, Validation, Data curation, Conceptualization. **Chao-Hui Duan:** Validation, Formal analysis, Conceptualization. **Hui-Ling Hu:** Visualization, Methodology, Conceptualization.

## Ethics approval and consent to participate

The study was approved by the ethics committees of the Sun Yat-sen Memorial Hospital, Sun Yat-sen University.

## Funding sources

National Natural Science Foundation of China (82471734, 82000803), Guangdong Basic and Applied Basic Research Foundation (2024A1515012990), Guangzhou Science and Technology Plan Project (202201010994), Guangdong Basic and Applied Basic Research Foundation (2024A1515011037).

## Declaration of competing interest

The authors declare that they have no known competing financial interests or personal relationships that could have appeared to influence the work reported in paper.

## Data Availability

Data will be made available on request.

## Abbreviations

NAAT: Nucleic Acid Amplification Tests; ddPCR: droplet digital PCR; CV: Coefficient of Variation.
